# Disturbed flow mediated modulation of shear forces on endothelial plane: A proposed model for studying endothelium around atherosclerotic plaques

**DOI:** 10.1038/srep27304

**Published:** 2016-06-03

**Authors:** Uma Maheswari Balaguru, Lakshmikirupa Sundaresan, Jeganathan Manivannan, Reji Majunathan, Krishnapriya Mani, Akila Swaminathan, Saravanakumar Venkatesan, Dharanibalan Kasiviswanathan, Suvro Chatterjee

**Affiliations:** 1 Vascular Biology Lab, AU-KBC Research Centre, MIT campus of Anna University, Chennai, India; 2 Centre for Biotechnology, Anna University, Chennai, India

## Abstract

Disturbed fluid flow or modulated shear stress is associated with vascular conditions such as atherosclerosis, thrombosis, and aneurysm. *In vitro* simulation of the fluid flow around the plaque micro-environment remains a challenging approach. Currently available models have limitations such as complications in protocols, high cost, incompetence of co-culture and not being suitable for massive expression studies. Hence, the present study aimed to develop a simple, versatile model based on Computational Fluid Dynamics (CFD) simulation. Current observations of CFD have shown the regions of modulated shear stress by the disturbed fluid flow. To execute and validate the model in real sense, cell morphology, cytoskeletal arrangement, cell death, reactive oxygen species (ROS) profile, nitric oxide production and disturbed flow markers under the above condition were assessed. Endothelium at disturbed flow region which had been exposed to low shear stress and swirling flow pattern showed morphological and expression similarities with the pathological disturbed flow environment reported previously. Altogether, the proposed model can serve as a platform to simulate the real time micro-environment of disturbed flow associated with eccentric plaque shapes and the possibilities of studying its downstream events.

Shear stress (SS) is a blood flow associated frictional force that regulates many vascular functions such as maintenance of acute vessel tone, vascular permeability, adhesion of leukocytes, development of blood vessels, secretion of prothrombotic and antithrombotic signaling molecules^1^,^2^. Variations in shear stress define endothelial phenotype. Vascular pathologies such as atherosclerosis, thrombosis, and aneurysm are developed as a result of disturbed blood flow^3^,^4^. Local flow around the atherosclerotic micro-environment modifies the endothelial phenotypes, which leads to the initiation and development of plaque^5^. It also plays key role in transformation of a stable plaque into a vulnerable plaque^6^. In order to explore the shear stress associated changes in endothelial cells (ECs) several *in vivo* and *in vitro* models were developed. Different approaches such as naturally occurring flow-disturbed regions, surgical interventions in animals, vertical step flow chamber, modified cone-and-plate viscometer and microfluidic model systems have been taken account to elucidate the phenotypic adaptation of ECs under varying flow conditions^3^,^7^. However, these models have their own limitations such as high cost, complicated technique, smaller sample size and difficult surgical manipulation.

Flow systems that can overcome drawbacks of existing models and facilitate exposure of ECs to controlled multidirectional flow environments along with the opportunity of co-culture with other cells would be useful to study mechanistic insights of plaque growth and rupture in atherosclerotic micro-environments. Hence, we made an effort to develop a simple, inexpensive and versatile model to generate disturbed flow. Based on computational fluid dynamic (CFD) predictions we have designed a model which can produce disturbed flow at the plaque site. Harvesting cells from defined flow regions for molecular and cellular studies is a significant feature of this model. This unique flexibility and advances are not attainable from the currently available shear stress models. In present study, we have also demonstrated the differential adhesion properties of endothelium around the blocks under different shear stress pattern that emphasizes the suitability of this model for cell to cell interaction studies in atherosclerotic micro-environments. Furthermore, the phenotypic differences of ECs identified under different flow regions can be used to correlate their pathological condition such as inflammation, plaque formation and thrombosis. Finally, different shape of blocks used in this model portrays the versatility of this model and its usefulness for elucidating the influence of geometrical shapes of the plaques in the event of plaque rupture and thrombosis.

## Materials and Methods

### Materials

#### Cell line

The human endothelial line EAhy926 was obtained as gifted from Dr. C.J.S. Edgell, Tissue Culture Facility, UNC Lineburger Comprehensive Cancer Center, and University of North Carolina. The cells were cultured in DMEM supplemented with 10% FBS (v/v) and 1% penicillin (w/v) and streptomycin (w/v).

#### Chemicals

Dulbecco’s modified Eagle’s medium (DMEM) and Fetal bovine serum (FBS) was purchased from PAN-Biotech GmbH (Am Gewerbepark, Aidenbach). Green-fluorescent Alexa Fluor 488 dye purchased from Molecular probes, Invitrogen. 2′,7′-dichlorofluorescein diacetate (DCF-DA), Di-hydro Rhodamine (DHR) from Invitrogen (Bangalore). 4′,6-Diamidino-2-Phenylindole, Dihydrochloride (DAPI), Amplex red Diaminorhodamine-4M-AM (DAR-4M-AM), were purchased from Sigma Chemical Co (St. Louis, MO) USA. Annexin V and PI staining kit was purchased from Abcam. All other chemicals used in this study were of reagent grade and obtained commercially.

### Methodology

#### CFD Simulation and wall shear stress calculations

CFD simulations were performed using the commercial software ANSYS FLUENT. 2D mesh for laminar and disturbed flow was generated and analysis were done with parameters described by Szymanski *et al.* 2008^8^. For boundary conditions, no-slip boundary conditions, velocity inlet (0.2 m/s) and pressure outlet were utilized. Water was used as working fluid (a homogeneous, incompressible Newtonian fluid; with *ρ* = 998.2 kg/m^3^, *μ* = 1.03 × 10^−3^ kg/m·s). All simulations were performed by using an iterative, segregated solution method until a converged solution was obtained. Navier-Stokes simulation was carried out using the FLUENT software^9^. In order to produce disturbed flow, the same geometry with different shapes of blocks; circle, square, triangle and irregular shapes were placed in the path of flow and run for FLUENT analysis with the same parameter used for laminar flow. The shear stress exhibited on the cells was calculated as a function of the measured flow Q from the following formula in parallel plate flow chamber (PPF):


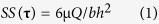


where μ = fluid viscosity (0.01 dyne second/cm^2^) b = width of the chamber (2.1 cm), h = distance between plates (0.019 cm). For the present study we have generated 2D mesh, which precisely mimics the 2D planar surface of parallel plate flow system and from that we obtained velocity profile over the planar surface. Shear stress (SS) was then extrapolated from velocity profile^10^ using the following equations,


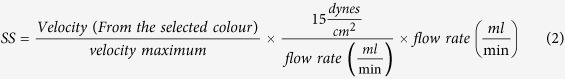


Flow rate kept constant so,


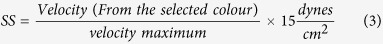


We have taken the average of velocity from DS1/2/3 (around 50 points) and substituted to the following formula (2 & 3) to obtain shear stress.

#### Fabrication of disturbed flow shear stress apparatus (DSSA)

Parallel plate fluid flow chamber was used to study the metabolic responses of endothelial cells *in-vitro*. In the present study, we have created disturbed flow based on CFD simulation done on 2D mesh created for parallel plate flow chamber. A circular block with a diameter of 0.6 cm and 0.02 cm height placed at the center. The height of circle kept same as that of the height between plates to ensure that the block is not misplaced when the flow is present and also it ensures that, the glass slide with coverslips do not break because of too much stress. EAhy926 cells grown on gelatin coated coverslips for overnight and placed upside down on the PPF chamber. A transparent cover was placed above the coverslip with cells then clamped on the sides to ensure the leakage proof. The PPF chamber with cells was then placed on 37 °C hot plate to ensure the physiological temperature *in vitro*. This setup was then connected with the inlet and outlet tube for running fluids. Shear stress running apparatus consists of five major parts 1) flow controller 2) Parallel plate flow chamber 3) peristaltic pump 4) upper buffer reservoir and 5) lower buffer reservoir. Media was loaded in the upper buffer reservoir enters the flow controller because of the gravitational force and from there it enters the Parallel plate flow chamber. Media passed through the flow chamber was collected in the lower reservoir. Peristaltic pump was used to pump the buffer back to the upper reservoir. Thus continuous flow for 30 min was achieved in this set up.

#### Naming and procurement of cells for experimentation

Based on the CFD simulation, regions where high and low shear stress were seen termed as following: Area 1 (DS1- disturbed shear stress region 1) – region in the front end of the block and the shear stress relative to the baseline level in straight vessels. Area 2 (DS2) – region in the other side of the block and the SS was low and retrograde flow was present. Area 3 (DS3) – region above and below the block, the SS was high, relative to the baseline level in straight vessels during increased flow velocity. Laminar flow with no block used to generate normal shear stress (NSS) and it served as control. ECs were subjected to 30 min of disturbed flow and laminar flow. Examination of the cellular, biochemical and molecular alterations were done on these specific regions of disturbed flow and compared with NSS and static controls.

### Validation of disturbed flow implications at cellular level

#### Cell counting and apoptosis

EAhy926 cells were grown on the coverslip overnight to attain optimal confluency (seeding- 1 × 10^5^–1 × 10^6^ cells per slide as per experimental requirement). Then the cells were placed upside down on the parallel flow chamber and normal and disturbed shear given for 30 min. After shear stress treatment, cells were washed with 1X PBS twice and bright field images were taken and were counted manually from the images. For the detection of apoptotic and proapoptotic cells, Annexin V and propidium iodide (PI) staining was used. Cells were subjected to normal and disturbed shear stress for 30 min. Then washed with 1X PBS twice then kept at 37 °C for 2 hrs for the observation of proapototic and apoptotic cells in all three regions. After 2 hrs, cells were incubated with binding buffer for 10 min followed by incubation with Annexin V and PI for 10 min. Fluorescent images of annexin and PI were captured using Olympus IX71 fluorescence microscope. Annexin V positive and PI stained cells were counted manually from the images.

#### Actin staining

EAhy926 cells were grown to 70–80% confluence and subjected to disturbed and normal shear stress for 30 min. Then washed gently with 1X PBS and fixed with 2% paraformaldehyde for 10 minutes. Then the cells were treated with 0.1% Triton X −100 for 1 minute for permeabilization and then incubated with 5 μM phalloidin (Green-fluorescent Alexa Fluor 488 dye). The fluorescent images were captured under Olympus IX71 epifluorescence microscope system equipped with a DP71 camera.

### Cell migration assays under disturbed flow

#### Trans well migration assay

Disturbed flow was created as previously mentioned. Cells from the selected regions were scraped and seeded on the upper chamber in equal numbers. The lower chamber was loaded with 10% DMEM media. The collagen coated membrane was placed between the upper and lower chamber on which the cells attach and migrate from the upper chamber to lower chamber side. The complete setup was incubated at 37 °C for 2 hrs in a CO_2_ incubator. After incubation, the membrane was carefully taken and wiped on the other side to ensure the presence of cells only on migrated side. Then the membrane with cells was fixed with 2% paraformaldehyde for 10 min. After fixation, the membrane was stained with DAPI for 10 min. Images were taken using Olympus IX71 epifluorescence microscope at 4X magnification. Cell migration was assessed by counting the cells on each well.

#### Wound Healing Assay

Scrape wound was made using sterile tip on endothelial cells monolayer subjected to 30 min of disturbed flow at DS1, DS2, and DS3 regions as a round wound. For every two hours interval images were taken using Olympus IX71 epifluorescence microscopy (4X magnification). Images were processed in ImageJ software to measure the reduction in wound area.

### Live cell tracking under disturbed flow

In order to monitor the morphological adaptations of ECs after exposure to disturbed flow from DS1, DS2 and DS3 regions cells were monitored under microscope for 2 hrs. EAhy926 cells were grown to 70–80% confluence and subjected to disturbed and normal shear stress for 30 min. then the cells coverslip with cells were kept in live cell chamber at 37 °C supplied with 5% CO_2_ for 2 hrs under microscope. Time-lapse images were taken at every10 min interval for 2 hrs using Olympus IX71 epifluorescence microscope at 20X magnification.

### Biochemical assays for oxidative stress measurements

#### Total ROS detection by Dichlorofluorescin diacetate (DCFDA)

Normal and disturbed shear stress was given for 30 min. Cells were washed with 1X PBS and incubated with DCFDA (10 μM concentration) for 10 min at 37 °C. Fluorescence image were taken and intensity calculation was performed using Adobe Photoshop version 7.0.

#### H_2_O_2_ measurement by Amplex Red

After 30 min of disturbed flow the cells were scraped from the three different areas under disturbed flow and incubated with 10 μM of Amplex red and 0.5 U of HRP for 15 min, and absorption was read at 570 nm.

#### Superoxide measurement by NBT

Normal and disturbed shear stress was given for 30 min. Cells were scraped from DS1, DS2, and DS3 regions and incubated with 1 mg/ml Nitro Blue Tetrazolium (NBT) for 2 hrs. After incubation, the medium was aspirated and the cells were washed with PBS twice. Formazan crystals formed due to the action of superoxides on nitroblue tetrazolium were dissolved by adding 200 μl of DMSO and the optical density was measured at 540 nm using a spectrophotometer (Varian Cary 4000 UV-Vis photometer, Varian, Ca, USA).

#### SOD activity measurement by Pyrogallol Assay

Cells were harvested from the disturbed flow regions and centrifuged at 2500 rpm for 5 min. The pellet was then homogenized in 500 μl of 20 mM Tris-Hcl buffer (PH 7.3) and centrifuged at 4000 rpm in 4 °C for 10 min. 75 μl of supernatant was boiled for 20 min and to that 365 μl of Tris-Cl (50 mM; PH-8.3) and 0.2 μM of Pyrogallol were added. This served as blank. To the 75 μl of supernatant, 365 μl of Tris-Cl (50 mM; PH-8.3) and 0.2 μM of Pyrogallol were added and incubated at 15 min in room temperature. After incubation, readings were taken at 420 nm. The absolute values were obtained by subtracting the blank values. Protein estimation in the supernatant was done using Nanodrop 2000 spectrophotometer (Thermo Fisher Scientific) and the final absolute values were normalized for protein concentration.

#### Peroxynitrite measurement by DHR

Normal and disturbed shear stress was given for 30 min. Cells were incubated with DHR at 10 μM concentration for 20 min at 37 °C. Fluorescence image was taken and intensity calculation was performed using Adobe Photoshop version 7.0.

#### Nitric oxide estimation using DAR 4M AM

Normal and disturbed shear stress was given for 30 min. Cells were washed with 1X PBS and incubated with 10 μM DAR 4M AM, fluorescent probe for 20 min, and then images were taken by Olympus IX71 epifluorescence microscope. Fluorescence intensity was calculated using Adobe Photoshop version 7.0 for NO production under both static and shear condition with respective control sets.

### Analysis of RBCs under disturbed flow

RBCs were isolated by spinning the blood at 3000 rpm for 20 min and washing 3 times with isotonic buffer. Disturbed flow was created as previously mentioned. Isolated RBCs were overlaid above the endothelial cells, and incubated at 37 °C in a CO_2_ incubator for 30 min. After incubation, coverslip with cells was gently washed twice with 1X PBS. Hematoxylin and Eosin staining was used to differentiate ECs and RBC. Images were taken by Olympus IX71 epifluorescence microscope. The RBCs overlaid on the cells were counted manually.

### Semi-quantitative RT-PCR analysis

Total RNA was isolated from DS1, DS2, DS3 regions cells and quantified using a Nanodrop 2000 spectrophotometer. Conversion of cDNA was performed on 200 ng of RNA using reverse transcriptase (Applied Biosystems). Primer sequences, annealing temperature and product size of RT-PCR analysis are summarized in Table 1. GAPDH was used as the house keeping internal control. The products were resolved in 2% agarose gel electrophoresis. The band intensity was quantified and plotted the graph. Cells treated with Phorbol 12-myristate 13-acetate (PMA) (15 ng/mL concentration) was used as control for the expression of inflammatory markers.

### Statistical analysis

All the experiments were performed in triplicates (N = 3) unless specified Data are presented as mean ± SD. One way ANOVA, student t-test and Turkey post-hoc tests were used to analyze the data. SIGMASTAT software package 6.0 was used for all statistical analysis and calculation of SEM values. P values ≤ 0.05 was considered as statistically significant.

## Results

### Establishing DSSA Model

**Figure 1:**
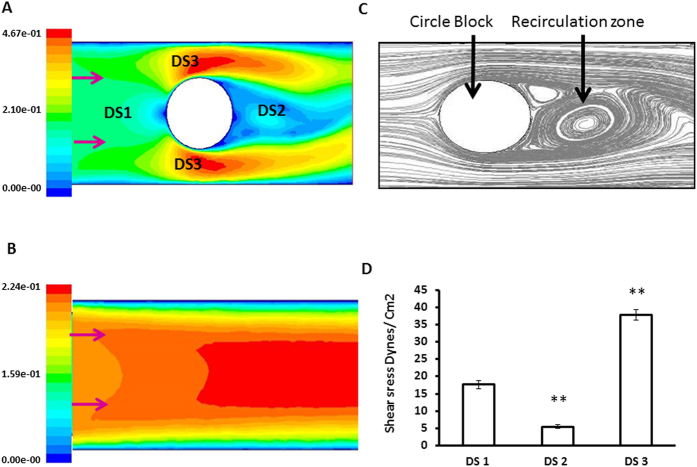
Results of CFD analysis. (**A**) Color contours shows the variations in velocity around the circle block. (**B**) Velocity profile represented in color contours for laminar flow (**C**) Path lines image showing the swirling flow and recirculation zone behind the circle block. (**D**) Wall shear stress around the block calculated from a CFD analysis based on the velocity represented as bar diagram. Graphs quantifying the average shear stress in DS1, DS2, and DS3 regions. The average velocity in region on the front end (DS1) was 0.23 m/s and shear stress was 17 dynes/cm^2^. Region at the other end (DS2) average velocity and shear stress was 0.07 m/s and 5 dynes/cm^2^ and 0.46 m/s and 34 dynes/cm^2^ at DS3 region. **P = <0.01 DS1 vs DS2 and DS1 vs DS3.

**Figure 2:**
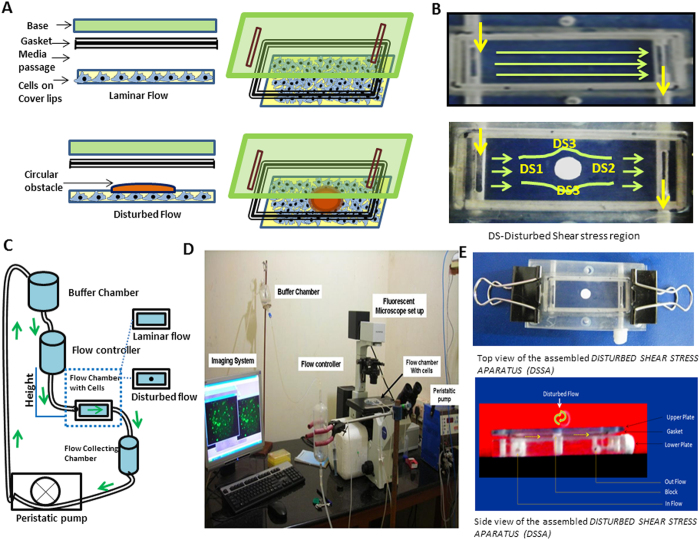
Pictorial representation of fabrication and working principle of DSSA. (**A**) Circular block with a width as that of the gasket placed at the laminar flow path to create disturbed flow around the block. Cartoon explains how circle block placed in PPF system to create disturbed flow (**B**) Image on the shows the top view of laminar flow; Image at the bottom shows the disturbed flow and regions selected for the study DS1, DS2, and DS3 based on the WSS measurement. (**C**) Shows the working principle of flow system. Flow setup consists of four main parts 1) Buffer chamber 2) flow controller 3) Parallel plate flow system (PPF) 4) Peristaltic pump. Silicon tubes connect the each part. Media flows through the PPF system collected in the lower reservoir and pumped to the buffer chamber with a use of peristaltic pump. (**D**) *invitro* flow apparatus (**E**) Top and side view of disturbed flow set up.

A two dimensional geometry of laminar and disturbed flow was generated and analyzed for velocity changes in ANSYS FULENT software. Figure 1A,B shows the difference in the velocity magnitude of laminar and disturbed flow and Fig. 1C shows the zone of recirculation behind the circular block. Results shows that the average velocity in the region on the front end (DS1) was 0.23 m/s and shear stress was 17 dynes/cm^2^. Region at the other end (DS2) the average velocity and shear stress was 0.07 m/s and 5 dynes/cm^2^. The average velocity and shear stress was 0.46 m/s and 34 dynes/cm^2^ in DS3 region. The path lines obtained from the simulation shows the swirling flow pattern at DS2 region where the velocity decreased drastically. The value of velocity in this region failed to induce physiological shear stress (Fig. 1D and Supplementary Fig. 1A). Normal flow (without obstacle placed at the center) velocity ranges from 0.1 m/s to 0.2 m/s and the average shear stress is 17 dynes/cm^2^ (Supplementary Fig. 1B,C). For the disturbed flow, DS1, DS2 and DS3 regions the average of velocity was taken as in Supplementary Fig. 2 and substituted in equation (3) to obtain shear stress. Using these data, we have developed a disturbed flow model as shown in Fig. 2A–E. The steps involved in fabrication of the apparatus were shown in Supplementary Fig. 3A–L.

### Validation of low and high shear stress effects at cellular level

**Figure 3:**
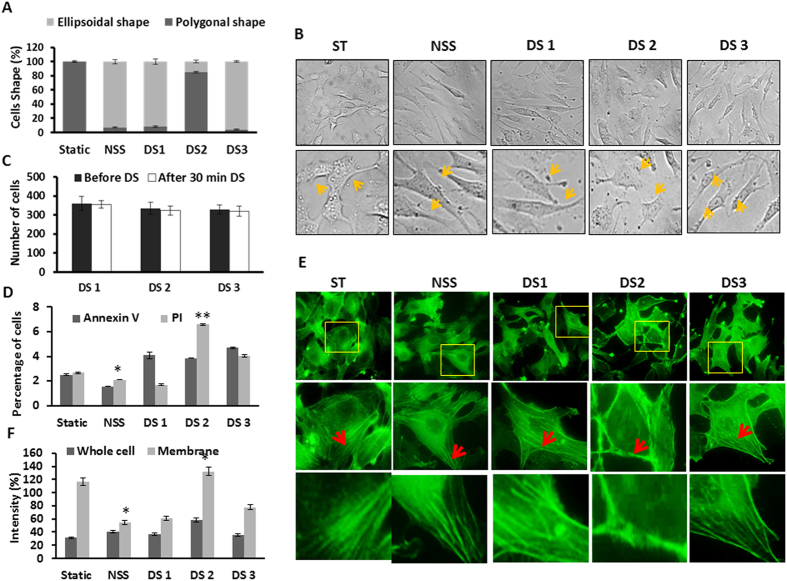
Morphological changes in endothelial cells exposed to normal and disturbed flow. (**A**) Graphical representation of cell morphology under disturbed flow. Cells elongated on the axis of flow in NSS, DS1 and DS3 regions. Cells in DS2 not attained elongated morphology rather maintained the polygonal shape (**B**) Panels of bright field images taken after 30 min of flow illustrates the cell morphology. Differences in shape are statistically significant (p < 0.05). (**C**) Graphs represent the cell counting before and after disturbed flow (p < 0.05). (**D**) Graphs represent the percentage of proapototic and apoptotic cells. Cells were stained with Annexin V and PI for proapototic and apoptotic cells detection. Significant increase in apoptotic cell population observed in DS2 region. *p = 0.01 Static Vs NSS; **p = 0.001 NSS Vs DS2. (**E**) Fluorescent images showing the highly polarized microfilaments in NSS, DS1 and DS3 cells and thick bands of actin at the periphery of cell in DS2 region (indicated with arrows points). (**F**) Organization of actin microfilaments under static, NSS and DS conditions. Cells were fixed after 30 min of NSS and DS and stained with phalloidin for actin microfilaments. Actin pattern under disturbed flow dispersed vs concentrated in periphery as whole cell vs membrane in graph. At DS2 region thick actin bands were seen at the periphery whereas centralized pattern seen in NSS and other DS regions. *P = <0.01 Static Vs NSS; NS Vs DS3.

In order to check the flow induced ECs morphological adaptation and alignment, elongation of cells in the direction of flow was observed. Evaluation of cell shape using image analysis of phase contrast imaging demonstrated that >80% of cells in NSS control, DS1, and DS3 regions showed ellipsoidal shape whereas in DS2 region, <20% of cells assumed an ellipsoidal shape (P = 0.05). Cells at DS1 and DS3 regions showed elongated morphology on the axis of flow whereas at DS2 regions the elongation was not seen in most of the cells (Fig. 3A,B), rather ECs in DS2 region maintains the polygonal shapes as that of the static control (Fig. 3B, DS2). This could be due to the presence of low shear stress and swirl flow in DS2 region. The cell number before and after disturbed was counted manually in all three regions. Results showed that the number of cells after disturbed flow was unaffected in all three regions (Fig. 3C). However, we could observe dispersed erosion of cells rather than at a particular place in DS2 region. Results of apoptotic and proapototic measurement showed that increased PI positive cells in DS2 region (Fig. 3D and supplementary Fig. 4A).

**Figure 4:**
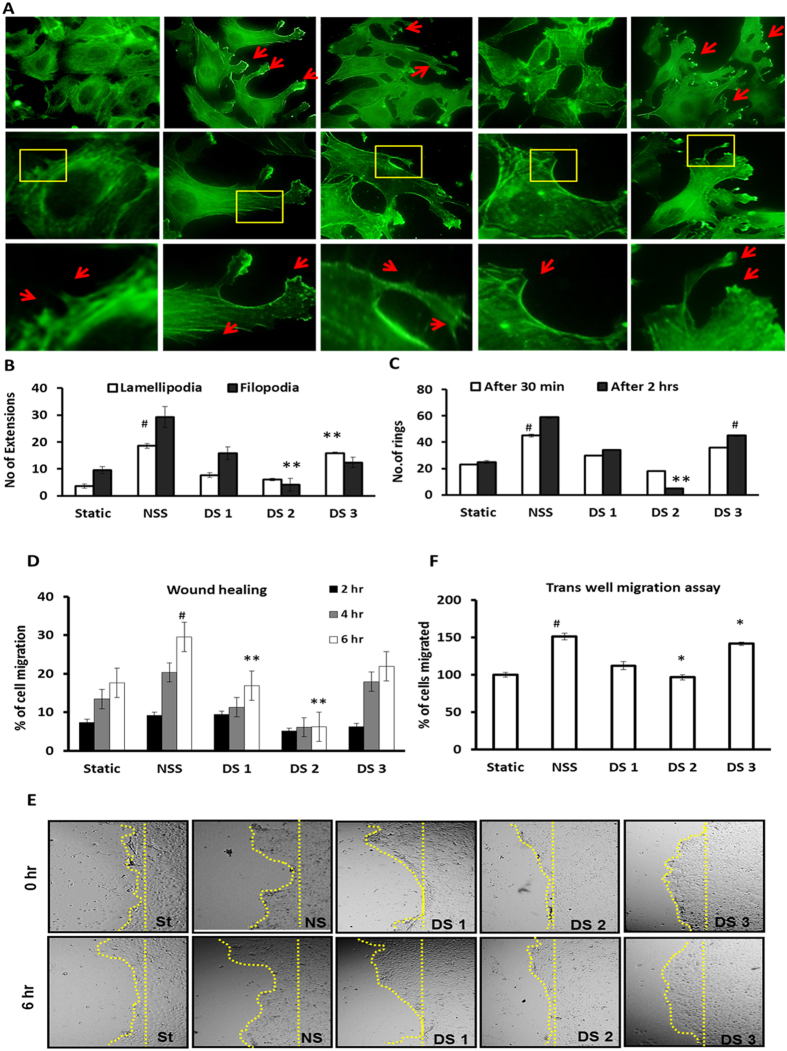
Absence of flow induced migratory structures and shear induced migration in low shear stress region. (**A**) Fluorescent image panels show the migratory structures after NSS and Disturbed flow. Arrow indicates the lamellipodia in top panels and filopodia in bottom panels. (**B**) Graphs represent the filopodia and lamelipodia formation under normal and disturbed flow. Flow induced migratory structures was observed more in NSS and other DS regions but not expressed by DS2 region where the shear stress was low. Lamellipodia formation **P = 0.001 DS1 Vs DS3; ^#^p = 0.03 Static Vs NSS; filopoidia **P = 0.001 NSS vs DS2. (**C**) Ring formation after 30 min of normal and disturbed flow was measured as a normal function of ECs. The number of rings formed at DS2 region immediately after 30 min of disturbed flow as well as after 2 hrs was significantly reduced at DS2 region where as NSS control and DS1 and DS3 regions rings formation increased. Ring **P = 0.001 NSS vs D2 2 hrs; ^#^p = 0.001 NS Vs DS3 2 hrs; Static Vs NSS 30 min. Endothelial cells subjected to normal and disturbed flow were analyzed for migratory capacity using (**D**) Scratch wound heling assay and (**E**) Bright field images show the comparison of reduction in wound area in Static, NSS, DS1, DS2, and DS3 region in scratch wound healing assay. (**F**) Trans-well migration assay. Decreased wound healing and the fewer migrated cells observed in DS2 region where the velocity of flow significantly low. Trans-well migration assay *p = 0.01 NSS vs DS3 6 hrs, and DS2 6 hrs, ^#^p = 0.05 Static Vs NSS 6 hrs. Wound healing. **P ≤ 0.001 NSS Vs DS2, NSS Vs DS1. ^#^p = 0.05 Static Vs NSS.

Sub-cellular polymerized actin pattern defines cell shape and plays important role in cellular functions such as locomotion, cell-cell interaction and signal transduction. Sub-cellular actin pattern changes rapidly and randomly during the blood flow, thereby the ECs align to the direction of blood flow. Under disturbed flow the polymerized actin has been reported to be present more at the center of cells than at the periphery^11^. In order to analyze the actin pattern, ECs were stained with phalloidin after disturbed flow. Results from the arrangement of polymerized actin demonstrated that the differential pattern of actin arrangement in DS2 region (Fig. 3E). Actin bundles were seen at the periphery of the cells in DS2 region which is opposed to the centralized dispersive pattern of actin in the other regions as well is in NSS control (Fig. 3F). Index of migratory structures can be taken as a measure of cells active state in the process of alignment under flow. Lamellipodia, the large migratory structures develop at the front end of a moving cell, and filopodia the micro-migratory structures were increased upon exposure to flow. Cells under no block (NSS) showed higher migratory structures. Cells in DS3 region compared to the other DS1 and DS2 regions possess more number of lamellipodia and filopodia (Fig. 4A,B) (P = 0.001). The absence of the lamellipodia and filopodia and reduced migration indicate the presence of disturbed flow in DS2 region.

In order to monitor the flow induced physical changes in ECs in DS1, DS2 and DS3 regions, they cell were monitored under microscope for 2 hrs in a live cell chamber after exposure to 30 min disturbed flow. In DS2 region, we could observe the detachment of cell-cell contact in 90 min whereas in DS1 and DS3 regions, cell-cell contact was intact (Supplementary Fig. 4B). Also, the number of endothelial rings (neighboring cells interact with each other and forms ring shape, which is the preliminary step in the formation of endothelial tubes) formed was reduced up to 10% in DS2 region, but increased to about 18%, 4% and 8% in NS control, DS1 and DS3 region (p = 0.001) which implies the loss of cell-cell contact in DS2 region (Fig. 4C).

ECs in the blood vessels are in quiescent state. A wound in the vessel induces the migration of endothelial cells. The healing capacity of ECs implies the healthy and active state of cells. Assays performed to assess the migration, such as scratch wound healing assay and trans-well migration assays revealed that the migratory capacity was affected because of disturbed flow. ECs from the DS1, DS2 and DS3 region showed 16%, 6%, and 21% healing respectively in scratch wound healing assay (Fig. 4D,E). This shows that among the three regions wound healing was significantly decreased in DS2 region (p = 0.05). Further results of the trans-well migration assay also showed that the cells from DS2 region showed reduction in cell migration about 55% when compared to NSS whereas only 39% and 10% reduction in migration were seen in DS1 and DS3 region respectively (Fig. 4F) P = <0.01.

### Modulation of Oxidative stress parameters and nitric oxide status

**Figure 5:**
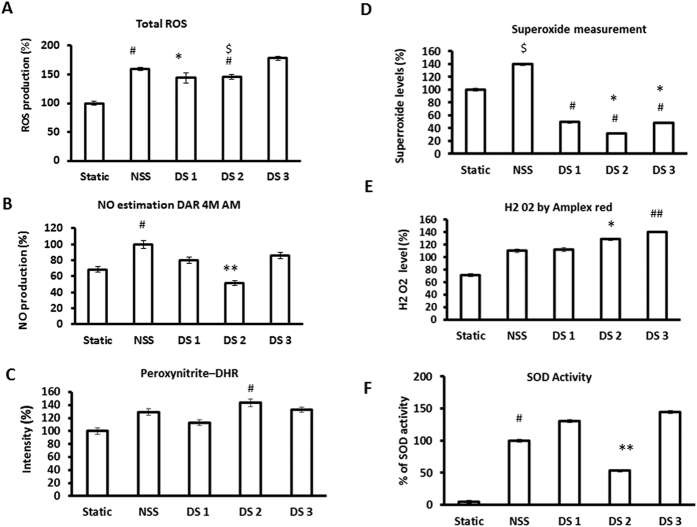
ROS validation under normal and disturbed flow. (**A**) Total ROS estimation was done using DCF fluorescent probe. Both normal and disturbed flow significantly increased the ROS production. ^#^p = 0.01 NSS Vs Static *P = 0.001 static Vs DS1; ^$^p = 0.01 NSS vs DS2. (**B**) NO estimation was done using DAR 4 M AM fluorescent probe. Cells in DS2 region where low shear stress observed NO production was low compared to the other DS regions. ^#^p = 0.05 NSS vs DS2; **P = 0.001 NSS Vs DS2. (**C**) Peroxynitrite measurement was done using DHR fluorescent probe. Significant increase in preoxynitrite production was observed in DS 2 region. ^#^p = 0.05 NSS vs DS2. (**D**) Measurement of Superoxide was done using Nitro blue tetrazolium. Superoxide levels were significantly reduced under disturbed flow and increased in NSS control compared to static control. ^#^P ≤ 0.005, *p = 0.01 Static Vs DS1, DS2, DS3; ^$^p = 0.01 Static Vs NSS. (**E**) Amplex red used for estimation of hydrogen peroxide. Both normal and disturbed flow significantly increased the H_2_ O_2_ production. P = 0.001 Static Vs DS3; P = 0.01 Static Vs DS2, DS1 Vs DS2. (**F**) SOD activity was measured using Pyrogallal assay. SOD activity increased in NSS, DS1 and DS3 region cells compared to static but only slight elevation seen in DS2 region cells compared to static. ^#^p = 0.01 Static Vs NSS; **P = 0.001 NSS Vs DS2.

The balance between the generation and removal of ROS is important for many cellular functions^12^. ECs at non-uniform, irregular, oscillated and recirculation zone in arteries shows oxidative stress^13^. Increased levels of ROS, including superoxide (˙O_2_−), hydrogen peroxide (H_2_O_2_), and ONOO^−^ at the disturbed flow region leads to dysfunction of ECs. ECs subjected to 30 min of disturbed flow and laminar flow produced high amounts of ROS compared to static controls (Fig. 5A). Total ROS production was increased about 43%, 45% and 78% in DS1, DS2 and DS3 regions respectively compared to static control (p < 0.01). Nitric oxide production under disturbed flow was assessed by DAR 4M AM. NO production by ECs under disturbed flow was reduced about 20%, 59% and 15% in DS1, DS2, and DS3 regions respectively compared to NSS control (Fig. 5B) p < 0.01. Peroxynitrite levels were increased about 13%, 43% and 32% in DS1, DS2 and DS3 regions respectively compared to static control (Fig. 5C) (p < 0.01). Superoxide levels were increased to about 40% in NS whereas reduced to about 51%, 69% and 53% in DS1, DS2, and DS3 regions respectively compared to static control (Fig. 5D). Hydrogen peroxide level was 63%, 73% and 79% in DS1, DS2, and D3 regions whereas 40% in static control (Fig. 5E). Superoxide dismutase (SOD) activity in these region measured by pyrogallol assay showed that increased about 30% and 44% in DS1 and DS3 respectively whereas decreased about 47% in DS2 regions compared to NSS control (Fig. 5F).

### Validation of low and high shear stress effects at molecular level

**Figure 6:**
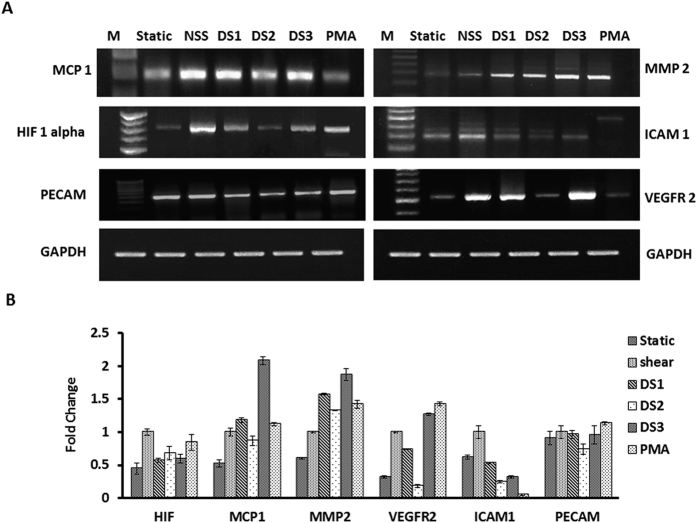
Semi quantitative PCR analysis of disturbed flow markers. (**A**) The mRNA expression levels of VEGFR2, PECAM-1, ICAM-1, HIF1 Alpha, MMP2 and MCP-1 were quantified using RT PCR and represented in panels. (**B**) The mRNA expression levels of VEGFR2, PECAM-1, ICAM-1, HIF1 Alpha, MMP2 and MCP-1 were measured by densitometry and presented as relative expression to housekeeping gene GAPDH in the bar graph. The expression levels seen under normal flow were normalized to a value of 1 as the standard for each factor. Differences in expression are statistically significant (p = <0.05).

Activated ECs produce chemokines, cytokines and adhesion molecules designed to interact with leukocytes and platelets, and target inflammation to specific tissues as a host defense mechanism^14^. EAhy926 cells subjected to laminar and disturbed flow was evaluated for expression of PECAM, VEGFR2, ICAM-1, MMP2 and MCP-1 mRNA levels (Fig. 6A,B). VEGFR2, a well-known shear associated molecule involved in the mechanotransduction via PECAM-VEGR2-VE cadherin mechanosensory complex was found to up-regulated in NSS control and DS3 region. There was a threefold increase in VEGFR2 expression observed in NSS laminar flow control and twofold and fourfold increased expression observed in DS1 and DS3 regions, but the increment in expression was not observed in DS2 region. ICAM and PECAM1 molecule are involved in adhesion of monocytes and other immune cells in atherosclerosis. There were two fold increases in ICAM-1 level in NSS compared to static. But this increment was not observed in disturbed flow regions. The expression level of PECAM was significantly decreased in ECs after exposure to disturbed flow whereas slightly upregulated in NSS control. MMP-2 is a matrix degradation enzyme playing a major role in cell migration. The presence of active MMP-2 implies the active role of ECs in shear mediated cell alignment. There were 2, 3, 2.5, and 3.5 fold increase in expression observed in NSS, DS1, DS2 and DS3 respectively. MCP-1 involved in the recruitment of leukocytes play significant role in the development of atherosclerotic plaque formation. There were 2,2 and 4 fold increase in expression observed in NSS, DS1, and DS3 region respectively. DS2 region showed less than 2 fold increment in MCP-1 expression. HIF1 alpha expression is used as marker for hypoxia state. There was less than two fold increased expression of HIF1 alpha observed in DS1, DS2 and DS3 region whereas two fold increases was observed in NSS control.

### Attraction of RBCs by differently sensitized ECs: a proof of efficiency of the DSSA model

**Figure 7:**
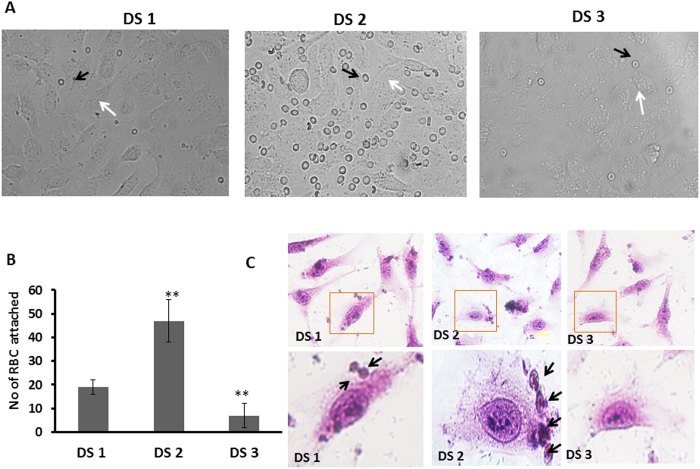
Adhesion of RBCs under disturbed flow. (**A**) Bright field images showing the RBC (indicated with black arrow points) adhered to ECs (white arrows). (**B**) Graphs represent the RBC adhesion to sensitized ECs. (**C**) ECs and RBCs stained with Hematoxylin and Eosin staining. Arrows points indicate RBCs adhered to ECs. **p = <0.05 DS1 Vs DS2 and DS3.

Increased platelet reactivity and coagulation are present in patients with atherosclerotic plaques which are not present in the intima of healthy arteries^15^. To check whether the EC differentially sensitized by the disturbed flow attract RBCs, we carried out an experiment with human RBC isolated from healthy individuals. RBCs were overlaid above the ECs exposed to disturbed flow and allowed to adhere for 30 min. After that a brief wash given with PBS and stained with Hematoxylin & eosin staining. Results revealed that DS2 region attracted high number of RBCs compared to DS1 and DS3 regions (Fig. 7A–C).

### Adaptability of DSSA for different shapes of block

**Figure 8:**
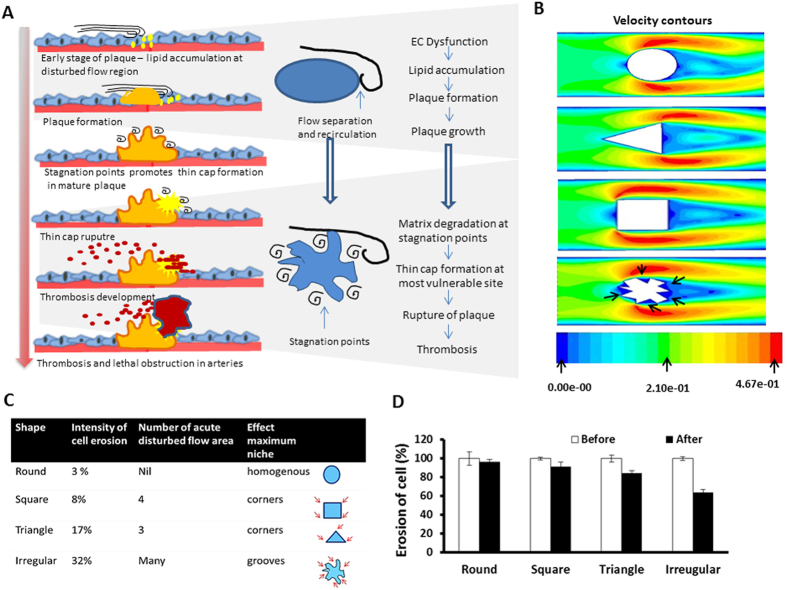
Future Scope of this model for studying the influence of plaque shape in plaque development, growth, and rupture. (**A**) Velocity magnitude and flow pattern gained from CFD analysis for different shapes. Low velocity and low shear stress observed in microgrooves of irregular shape (indicated using arrows). (**B**) Cartoon signifies the influence of shape on atherosclerotic plaque formation and rupture. Stagnation points at the latter plaque stages can be responsible for the thin cap formation and rupture. (**C**) Cell number was counted after placing different shapes in the DSSA. There was reduction in cell number observed when irregular shape placed (p = <0.05 before Vs after DS). The micro grooves in irregular shape were indicated using arrows. (**D**) Graph quantifies the cell detachment after 30 min of disturbed flow with different shape.

Recently many studies reported the importance of plaque architecture in thrombosis formation^5^. Studying impact of different shape on endothelial monolayer will be useful to understand the influence of shape of block in the formation of vulnerable plaques and thrombosis (Fig. 8A). To show the flexibility of DSSA for adapting different shapes, we have placed different shaped block instead of circle in DSSA. Velocity magnitudes for different shapes (square, triangle, and irregular) were obtained from CFD analysis. The regions proximal to the circle, corners of the triangle and square and the grooves in irregular shape velocity was <0.01 m/s shear stress were <1 dynes/cm^2^ (Fig. 8B and Supplementary Fig. 5A–C). In order to study the impact of different shape on endothelial monolayer these shapes were placed in DSSA. It was observed that significant reduction in cell number was seen in triangle (18%) and irregular (32%) shapes after 30 min of disturbed flow (Fig. 8C,D) (P = <0.05).

## Discussion

Disturbed shear stress is associated with vascular conditions such as atherosclerosis, thrombosis aneurysm etc. Endothelial monolayer cells are constantly exposed to shear stress which triggers the signaling events including opening of ion channels, modulation of gene expression and activation of several signaling pathways^16^. The shear stress activated signaling could be protective or distractive to the system depending on the type and magnitude of shear stress^1^. Therefore, it is crucial to understand the physiology of endothelial cells under varying flow conditions rather than static culture. In order to elicit the role of disturbed flow on ECs, previous studies have put fourth various *in vitro* and *in vivo* models^3^. However, the *in vitro* models that are in use have shown few disadvantages in various aspects^3^,^7^. Hence, a model that simulates disturbed flow environment with features to overcome some of the drawbacks in previous models could pave way for better understanding of disturbed flow associated vascular conditions. Hence, in the present study we have constructed a new disturbed flow model using parallel plate flow (PPF) apparatus and validated the model using biochemical, signaling and expression changes associated with disturbed flow.

In the present model we could able to produce physiological shear stress and re-circulatory flow with low shear stress within the same monolayer of endothelial cells. Disturbed rheology around the atherosclerotic plaque has been postulated by many computational simulation models studies^17^,^18^,^19^. Recently, Assemat *et al.*^20^ showed the recirculation zone was generated behind the plaque by local flow around the plaque^20^. In this model, we simulate plaque like athero-prone environment by using a circular block which creates the recirculation zone (DS2 region) as seen around the plaque. The results of CFD analysis showed the zone of recirculation (Fig. 1C) and low shear stress (<5 dynes/cm^2^) region, which is similar to other computational predictions reported earlier. Also, this model has shown to overcome few important drawbacks of the existing models including 1. Less cellular detachment observed when compared with step flow model; 2. No wave like uneven flow pattern and evaporation as seen in cone plate model; 3. Collection of large number of cells is possible for downstream analysis such as biochemical analysis and macromolecular isolation (RNA and protein) for expression assays which is less possible in commercially available miniature flow systems and micro devices^3^,^21^.

Further, validation of this model with (1) computational simulation, (2) cellular and (3) biochemical signaling are the optimal way to prove the efficiency of the model. In the cellular context, we observed morphological modulations of ECs after shear stress, was consistent with the differential shear stress patterns as observed in the simulation (Fig. 3A,B). The shape of EC is strongly affected by fluid shear stress and flow direction which initiates a cytoplasmic actin-remodeling response that contributes in filament alignment^22^. It is also evidence from the results of polymerized actin staining which shows thick actin bundles at the periphery of cells in DS2 region and a dispersed pattern in NSS, DS1, and DS3 region (Fig. 3E,F). Mengistu *et al.*^23^ showed that higher shear stress treatments uses JNK pathway for mechanotransduction from apical surface to the sub-cellular actin that culminates at 30 min after flow onset with an eight-fold increase in activity compared to that of static EC cells^23^. Further, shear stress dependent adaptation of EC resulting in rearrangement of filamentous actin (F-actin) into bundles of stress fibers, and align with the flow direction to form a diffuse network of short microfilaments including lamellipodia and filopodia^13^. A pioneering approach already explored that prominent central microfilament bundles and the reduced peripheral microfilaments are seen at localized regions experiencing elevated shear stress at branch sites and at the aortic bifurcation^24^. Consistent with the above observations, current observation (Fig. 3A,B) have also shown that our model efficiently mimic and visualize the effect of normal and disturbed flow on directional alignment nature of EC. Visualizing the migratory nature of EC under the disturbed flow would be obvious to support the current model. Our previous study already demonstrated that, endothelial cells exposed to 15 dynes/cm^2^ shear stress promoted migration and migratory structure formation^11^. Also, earlier studies reported that, disturbed flow leads to non-uniform directionality and reduced directional persistence of ECs^3^. Area of disturbed flow leads to non-uniform and slower cellular migration along with reduced directional persistence^11^,^25^. Consistence with the above, current observation have shown that our model also mimic the above conditions at DS2 regions where disturbed and swirling flow occurs that also extends to migratory disparity of EC (Fig. 4A,B). The cells in the DS2 region are relatively less migratory with limited migratory extensions, while the ECs in the regions of laminar flow tend to behave more migratory (Fig. 4D,E).

It is evident that association of disturbed flow at branching sites increases susceptibility of endothelium to apoptosis and higher turnover^26^. Recently, a human study on the release of microparticles (CD62E and CD31^+^) and their characteristics indicates that disturbed blood flow creates a proatherogenic vascular endothelial phenotype by acutely inducing activation and apoptosis of the vascular endothelium^27^. Subsequent studies in similar direction demonstrated that disturbed flow induces oxidative stress and activation of protein kinases in endothelium^28^,^29^. Similarly, present study elaborates that the ECs under disturbed flow regions (DS2) are proapototic with weakened cell-cell contact (Fig. 4C and Supplementary Fig. 3A). Reactive oxygen species (ROS) production by vascular endothelial cell is associated with endothelial dysfunction under various circumstances^30^,^31^. Disturbed flow also increases apoptosis in ECs by promoting the production of ROS that reacts with NO to form peroxynitrite (ONOO^−^) which is also one of the consequences and cause of oxidative stress induces proatherogenic responses in ECs^28^,^29^,^32^. In the current observation, elevation of ROS level and SOD activity coupled with elevated levels of the peroxynitrite in DS2 region proves the mimicking reliability of the present model with the pathophysiological environment (Fig. 5A–F).

Further, signaling axis also demonstrated the ability of current model to accurately replicate normal and disturbed flow conditions by harvesting cells from all three regions (DS1, DS2 and DS3) and mRNA expression analysis of selective marker genes whose expression was dependent on flow as mentioned in earlier studies. VEGFR2, one of important mechanotransducer mediates many signaling mechanisms through production of nitric oxide under the influence of shear stress^33^. Recently, works of Eleni Tzima1 *et al.*^34^ and Coon *et al.*^35^ portrayed the role of VEGFR2 signaling in shear stress through PECAM-VEGFR2-VE cadherin mechanosensory complex. The work of Hasan Erbil Abaci *et al.*^36^ showed that *eNOS* and *VEGFR2* levels were downregulated under low shear stress and O_2_ tension and provided the evidence for the inhibition of angiogenesis in ischemia. It is also proven from the results of the expression analysis which showed significant reduction of VEGFR2 and PECAM-1 in low shear stress region (DS2) at the same time increased expression of VEGFR2 was seen in DS3 (Fig. 6A,B). Similarly, monocyte chemoattractant protein-1 (MCP-1/CCL2), one of the key chemokines, regulates migration and infiltration of monocytes^37^, and the regime expressed transiently on the flow onset irrespective of the flow type^3^,^38^. In present experimental set up, MCP1 expression was observed in NSS control and disturbed flow regions whereas the MCP1 expression was not consistent with previous studies. The work of Milkiewicz *et al.*^39^ and Yamane *et al.*^40^ showed that MMP-2 mRNA and protein levels in cultured microvascular endothelial cells were significantly low in response to shear stress exposure whereas EC activated by low shear stress or disturbed flow showed increased expression of the MMP-2. In the present model, all the three regions (DS1, DS2, and DS3) increased expression of MMP2 in ECs as observed compared to laminar control suggesting that differential modulation of matrix degrading components occur when a block placed in the laminar path. Generally ROS has been shown to activate MMP-2, a key protease in cardiovascular diseases. A study by Belkhiri *et al.*^41^ on hydrogen H_2_O_2_ induced MMP2 role in degradation of adjacent basement membranes showed that oxidants such as H_2_O_2_ stimulates the expression of MMP and influences the remodeling of vascular basement membranes by endothelial cells. In another study Ali *et al.*^42^ showed that treating cardiomyocytes with H_2_O_2_ led to elevated MMP-2 level/activity. Also the work of Zhang HJ *et al.*^43^ showed that overexpression of MnSOD in human breast cancer MCF-7 cells with plasmids containing MnSODcDNA stimulated the activation of MMP-2 with a corresponding elevation of ROS. The increment in MMP2 level under disturbed flow in our study could be the influence of increased H_2_O_2_ and superoxide dismutase activity under disturbed flow.

Further expansion of scope of the present model RBCs were overlaid on the endothelium subjected to disturbed flow regions that describes the differential pattern of RBC-endothelium attraction based on the nature of flow around the plaque. Enhanced cell- cell interactions of platelets, leukocytes, and red blood cells in low shear stress regions and release of ADP (platelet agonist) a well-known activator of platelet aggregation at high shear rates and high hematocrits regions, are indications of definite role of fluid shear-stress in formation of thromboembolism^44^,^45^. RBC attaraction to endothelium is a critical hematologic factor in several physiological and pathological milieus. Increased RBC attraction was first demonstrated in sickle cell disease. Mechanistic insight of the events has been proposed including several endothelial and RBC adhesion molecules such as VCAM-1, α4β1, Lu/BCAM, ICAM-4. In malaria, Plasmodium falciparum erythrocyte membrane protein1 sticks to ICAM-1, PECAM-1 and augments the parasite dissemination. In diabetes mellitus higher RBC adhesion is correlated to the severity of vascular complications. However in physiological conditions the negative charges in the glycocalyx of red blood cells (RBC) and vascular endothelial cells (EC) facilitate frictionless blood flow through blood vessels. Recently, many studies reported the involvement of impaired glycocalyx volume in in plaque development and thrombotic events^46^. Also, it is evident from Hans Oberleithner *et al.*^47^ study on EC-RBC interaction, that when the RBC is brought in contact with the endothelial cell surface for a time period of 1 s, a significant retraction force is necessary to detach the RBC from the EC surface. From these studies it is evident that RBCs can attach to endothelial cells at low shear stress regions and can contribute to thrombotic events. Attraction of RBCs to susceptible ECs in DS2 region (Fig. 7A,B) reveals that the model can be adapted to study the thrombosis formation in real time. Thus, the model developed provides opportunity to study various aspects of plaque environment in vascular niche.

In the computational axis, based on the intense literature survey, we believe that this is the first study attempts to simulate and demonstrate the importance of plaque shape in influencing the local flow through an *in vitro* model. With few modifications with different shaped blocks, this model can be employed to study the eccentric nature of flow around atherogenic plaque. Existing simulation and numerical model considered the plaque at macro levels and ignored the micro pocket regions in the complex geometry of plaque. It was evident from the present study that, the regions proximal to the circle, corners of the triangle and square and the grooves in irregular shape the shear stress were <1 dynes/cm^2^, which suggests the significance of shape of the block in microscale Fig. 8B and Supplementary Fig. 4A–C. Particularly, the micro-pockets zones in irregular shaped block (Fig. 8B and Supplementary 4D) velocity was close to zero and also more number of cells were detached when irregular shape block placed in DSSA. These preliminary data provided signifies the importance of plaque architecture in the development of vulnerable plaques (Fig. 8B) and also the impact of the current model.

## Conclusions

Endothelial cells, by virtue of their location in the vessel wall are constantly exposed to varying mechanical stress^48^. Hence, studying endothelial cells at varying flow pattern is appropriate to give mechanistic insights into many cardiovascular diseases^3^. Considering the limitation of the other models, we have developed the disturbed flow model with the following features 1) modifiable flow pattern in inexpensive way 2) Harvesting of cells from different shear region for molecular studies 3) studying single cell under varying flow pattern 4) Possibility of co-culture of other cells without much complication. Thus the model can be utilized as a prototype for studying the effects of disturbed flow interaction of blood cells at disturbed flow regions. Also, real plaque mimicking condition can be created by coating the different shapes of block with foam cells. Thus it provides efficient platform for studying mechanic insights of diseases and the cardio protective drug screening. Comparison of DSSA with existing model was given in Table 2.

It is evident from the red blood cell interaction experiments that this model can be employed to study the influence of blood cells like RBCs, and platelets on plaque disruption. Immune profile of adjacent ECs of the plaques can be monitored in real time by placing a surgically removed plaque rather than using sterile teflon blocks as used in the present study. Hence, the model possesses futuristic scope for researchers to study the cell-cell interaction and cell – plaque interactions, thus emphasizing the importance of this model. However, this model possesses limitations such as absence of stretching and pulsatile flow which is a requisite to replicate scrupulous *in vivo* conditions. In conclusion, we propose a model that is simple, inexpensive and easy to set up possessing advantages including studying disturbed flow effects on ECs.

## Additional Information

**How to cite this article**: Balaguru, U. M. *et al.* Disturbed flow mediated modulation of shear forces on endothelial plane: A proposed model for studying endothelium around atherosclerotic plaques. *Sci. Rep.* **6**, 27304; doi: 10.1038/srep27304 (2016).

## Supplementary Material

Supplementary Information

## Figures and Tables

**Table 1:** Semi-quantitative RT-PCR primers sequences and annealing temperatures.

S. No.	Gene	Primers	Annealing temperature	Reference
1.	GAPDH	Fwd- 5′-GGTGAAGGTCGGAGTCAACGGA-3′	56 °C	49
		Rev- 5′-GAGGGATCTCGCTCCTGGAAGA-3′		
2.	VEGFR2	Fwd- 5′-GTGACCAACATGGAGTCGTG-3′	63.4 °C	50
		Rev- 5′-CCAGAGATTCCATGCCACTT-3′		
3.	MCP-1	Fwd- 5′-CAGCCAGATGCAATCAATGC-3′	55 °C	51
		Rev- 5′-GTGGTCCATGGAATCCTGAA-3′		
4.	MMP-2	Fwd- 5′-TTTCCATTCCGCTTCCAGGGCAC-3′	56.1 °C	52
		Rev- 5′-TCGCACACCACATCTTTCCGTCACT-3′		
5.	HIF-1α	Fwd- 5′-GTCGGACAGCCTCACCAAACAGAGC-3′	55 °C	53
		Rev- 5′-GTTAACTTGATCCAAAGCTCTGAG-3′		
6.	ICAM-1	Fwd- 5′-AGGCCACCCCAGAGGACAAC-3′	61.3 °C	54
		Rev- 5′-CCCATTATGACTGCGGCTGCTA-3′		
7.	PECAM-1	Fwd- 5′-AGGGCTCATTGCGGTGGTTGTCAT-3′	55 °C	55
		Rev- 5′-TAAGGGAGCCTTCCGTTCTAGAGT-3′		

**Table 2:** Advantages and limitations of Disturbed flow model in comparison with other model.

Models/Parmeters	Cone plate	Step flow	Microfludics	Reciprocating flow	Animal models	DSSA	References
Microscopic analysis	+	+	+	+	+	+	3,7,56,57
Conditioned media collection	+	+	+	+	−	+	3,7,56,57
Macro Scale (Expression analysis by PCR/WB) of defined disturbed flow location	+	−	−	+	+	+	3,8,56,57
Co culture of other cells	+	+	+	+	+	+	3,7,56,57
Pulsatility/contractile function	−	−	+	−	+	−	3,7,56,57
Flexibility of changing shape	+	−	−	−	−	+	3,7,56,57
