# Supplementary material for: Disturbed flow mediated modulation of shear forces on endothelial plane: A proposed model for studying endothelium around atherosclerotic plaques
Source: Sci Rep. 2016 Jun 3;6:27304. doi: 10.1038/srep27304 (PMC4891674; doi:10.1038/srep27304)
Supplement: Supplementary Information [file srep27304-s1.pdf]

Disturbed flow mediated modulation of shear forces on endothelial plane: A proposed model for studying endothelium around atherosclerotic plaques

Uma Maheswari Balaguru[1], Lakshmikirupa Sundaresan[2], Jeganathan Manivannan[1], Reji Majunathan[1], Krishnapriya Mani[1], Akila Swaminathan[1], Saravanakumar Venkatesan[1], Dharanibalan Kasiviswanathan[2], Suvro Chatterjee[1, 2,*]

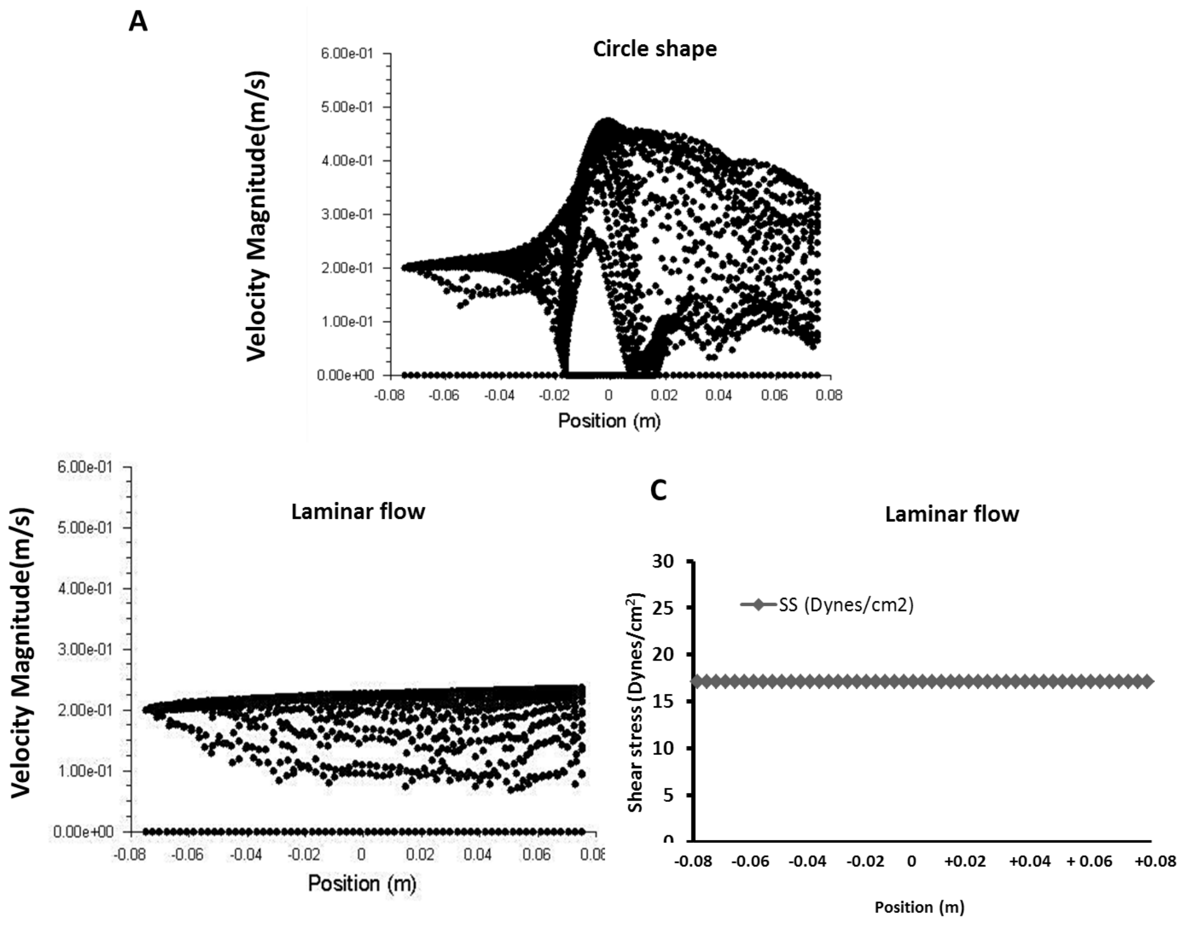

Supplementary Figure 1│ A) Velocity profile of Circle shape. Low velocity was observed in region behind the block (DS2), position 0 to +0.02m in graph B) Velocity profile of laminar flow and C) Calculated average shear stress in laminar flow.

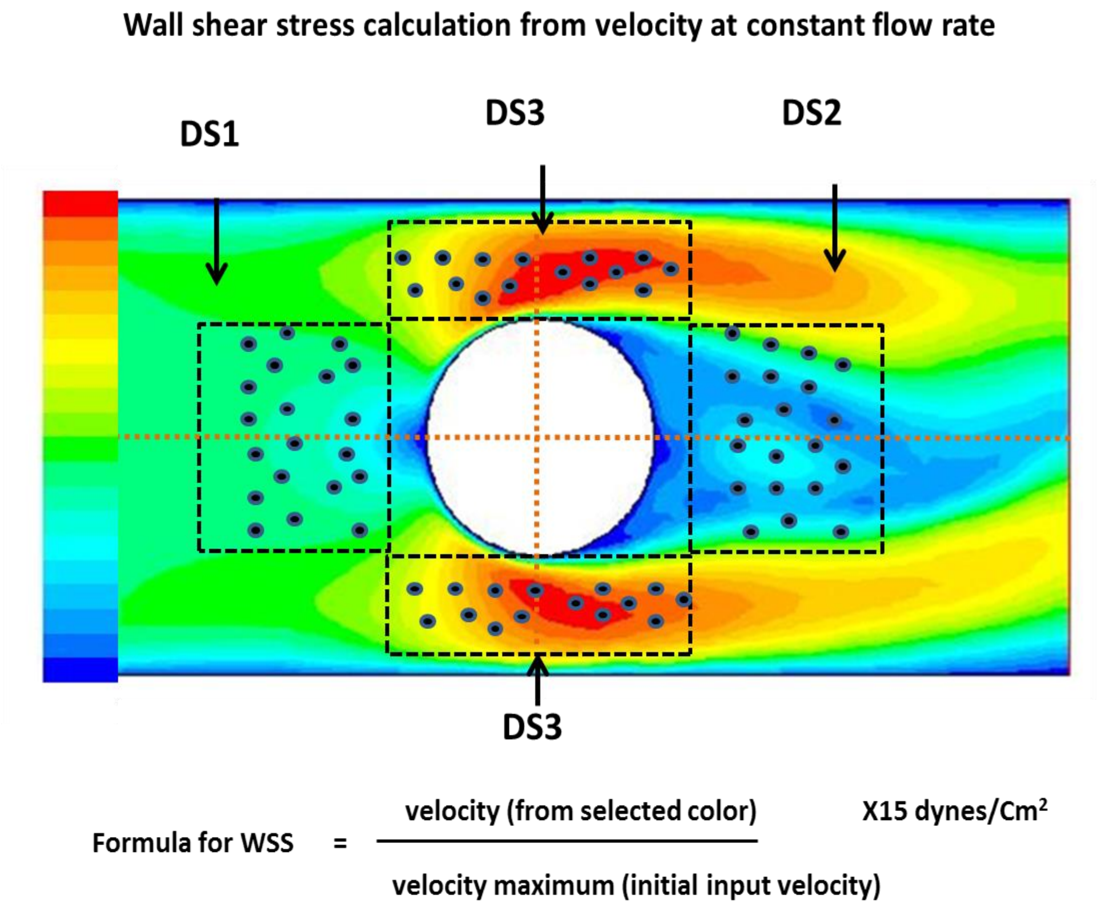

Supplementary Figure 2 | Image represents the different areas selected for the study (DS1/2/3) based on velocity profile and calculated shear stress.

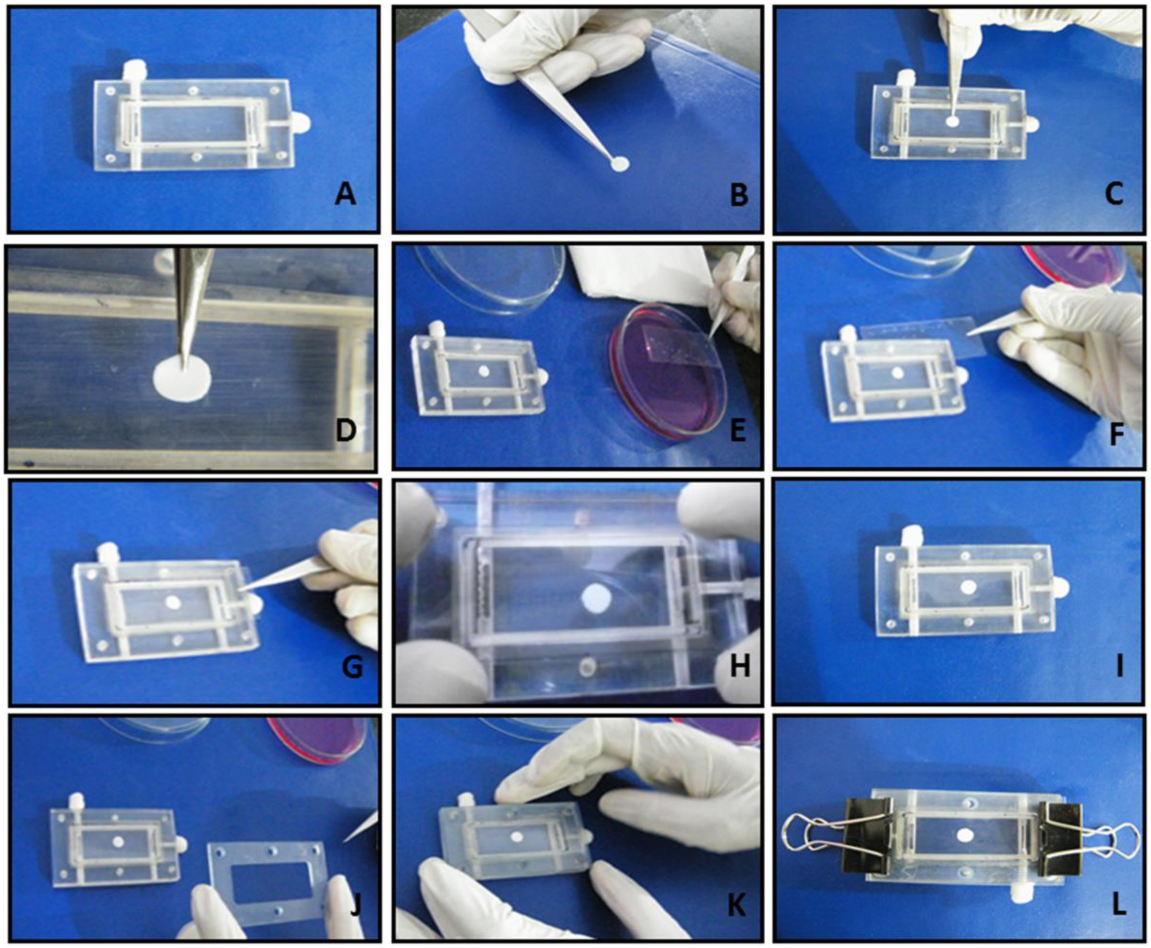

Supplementary Figure 3│ Depiction of steps in setting up the disturbed flow system A) Parallel plate flow system B) Circular block C) and D) Placement of block at the center of flow system (0.02cm width and 0.6 cm diameter) E) Cells grown on coverslips (24X60 mm) F, G, H and I) Placement of coverslips with ECs J) and K). Placements of transparent cover L) clamping to seal for leakage proof.

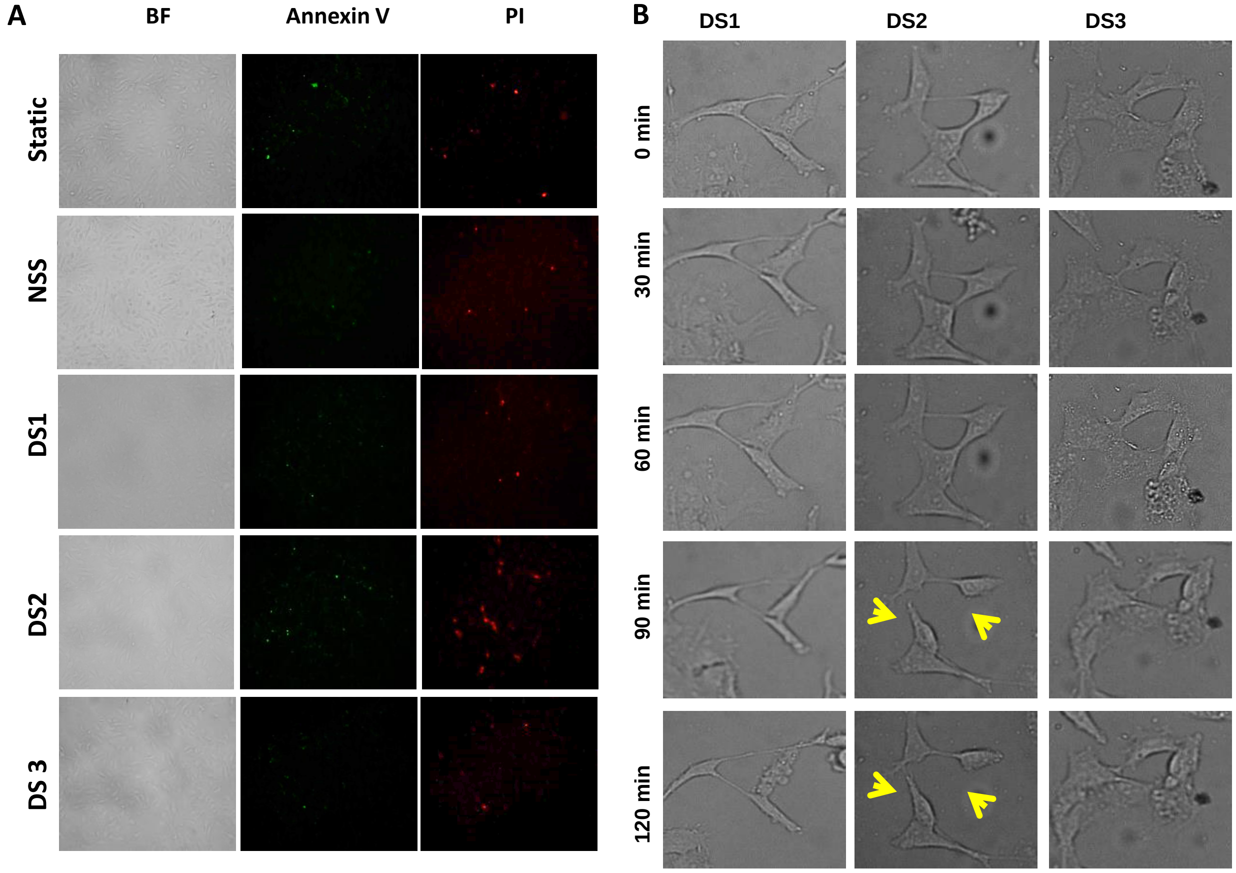

Supplementary Figure 4│ Low shear stress induced detachment of cell- cell contact and apoptosis. A) Panels show the time-lapse images. Cells from DS1, DS2 and DS3 regions were kept in a live cell chamber and observed for 2hrs after subjecting them for 30 min of normal and disturbed flow. Arrow indicates the detachment of cell-cell contact and rounding up of cells in DS2 region. B) Fluorescent image shows the of proapototic and apoptotic cells. Cells were stained with Annexin V (Green) for proapototic and PI (Red) apoptotic cells detection. Significant increase in apoptotic cell population observed in DS2 region. Left panel shows the bright field images, middle panel shows the Annexin V staining and Right panel shows the PI staining.

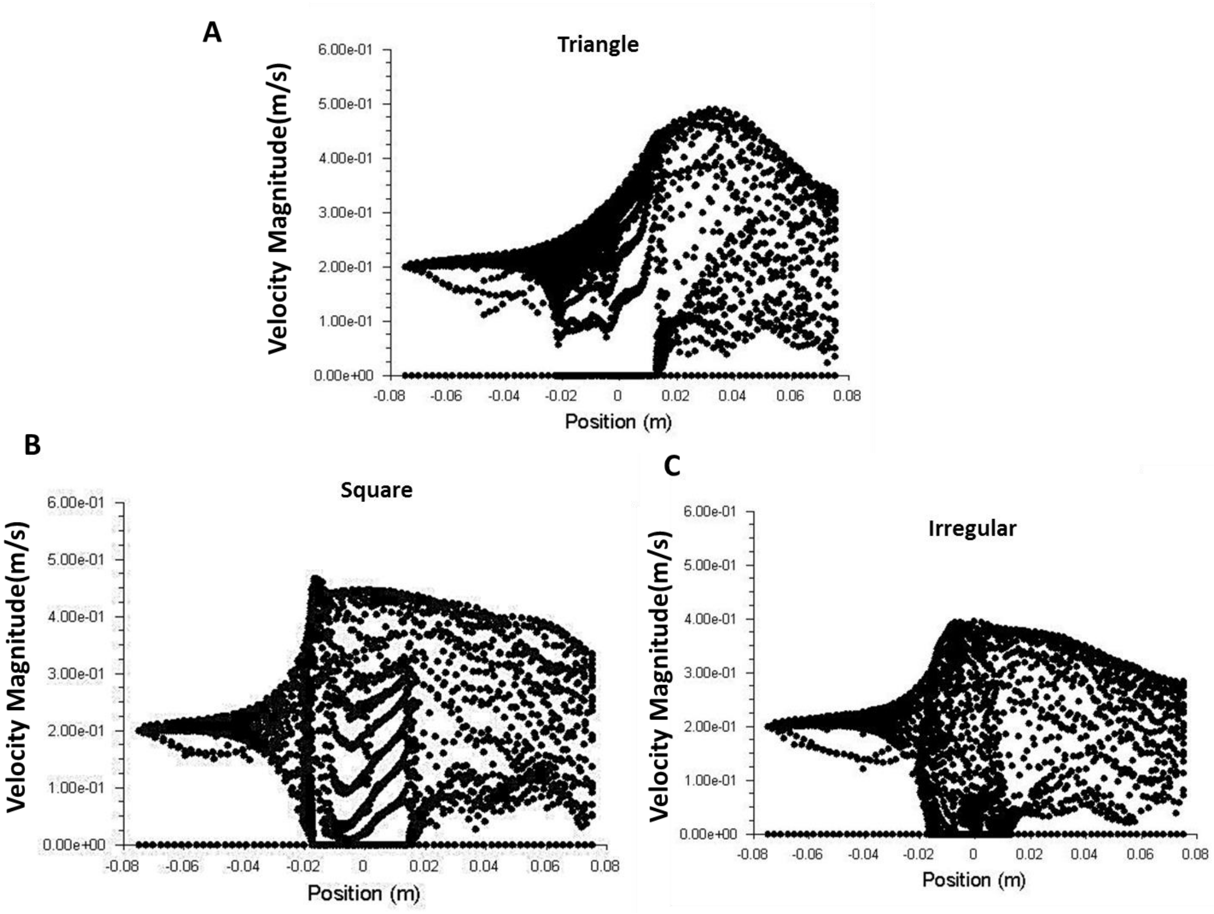

Supplementary Figure 5│Velocity profile of different shapes. A) Triangle B) Square C) Irregular shape.
